# Unleashing light's healing power: an overview of photobiomodulation for Alzheimer's treatment

**DOI:** 10.2144/fsoa-2023-0155

**Published:** 2024-05-24

**Authors:** Aakash Ramanishankar, Ankul Singh S, Rukaiah F Begum, Narayanan Jayasankar, Afreen Nayeem, Bhupendra G Prajapati, Shanmugasundaram Nirenjen

**Affiliations:** 1 Department of Pharmacy Practice, School of Pharmaceutical Sciences, Vels Institute of Science Technology & Advanced Studies, Pallavaram, Chennai. India; 2 Department of Pharmacology, SRM College of Pharmacy, SRM Institute of science & technology, Chennai, Tamil Nadu, India; 3 Department of Pharmaceutics, Anand College of Pharmacy Agra-Delhi Highway (NH2) Keetham, Agra, Uttar Pradesh, 282007, India; 4 Department of Pharmaceutics, Shree SK Patel College of Pharmaceutical Education & Research, Ganpat University, Kherva, 384012, India

**Keywords:** AD, ATP biosynthesis, beta-amyloid plaque, mitochondrial dysfunction, PBMT

## Abstract

**Aim:** Photobiomodulation involves the use of low-level light therapy or near-infrared light therapy found to be useful in the treatment of a wide range of neurological diseases. **Objective:** The aim is to review the mechanism and clinical applications of photobiomodulation therapy (PBMT) in managing Alzheimer's disease. **Methods:** To ensure that the consensus statement accurately reflects both the experts' viewpoint and the most recent developments in the field, the expert opinions were recorded and thoroughly reviewed. **Results:** PBMT elicits reduction of beta-amyloid plaque, restoration of mitochondrial function, anti-inflammatory and antioxidant properties with a stimulation in ATP synthesis. **Conclusion:** The PBMT could be helpful in patients non-responsive to traditional pharmacological therapy providing significant aid in the management of Alzheimer's disease when introduced into the medical field.

## Plain language summary

Alzheimer's disease (AD) is an incurable progressive neurodegenerative disease clinically manifested with a decline in cognitive function. To ensure that the consensus statement accurately reflects both the experts' viewpoint and the most recent developments in the field, the expert opinions were recorded and thoroughly reviewed. PBMT elicits various mechanisms such as reduction of beta-amyloid plaque, Restoration of mitochondrial function and maintenance the homeostasis, and anti-inflammatory and antioxidant properties with a stimulation in ATP synthesis. The PBMT could be helpful in patients who are non-responsive to conventional pharmacological therapy. This therapy might provide significant aid in the management of AD when introduced into the medical field. However, it requires various intensive research to be conducted for further conclusion.

## Abstract (plain language summary)

Photobiomodulation therapy (PBMT) was found to reduce the deposition and overload of beta-amyloid in the brain, especially in Alzheimer's disease (AD).

Mitochondrial dysfunction is one of the pathological markers involved in neuronal dysfunction that leads to the development of AD, which is characterized by impaired mitochondrial dynamics and mitochondrial fragmentation.

AD is the most acquired neurodegenerative disease and the major cause of depression and dementia in the elderly population, It is characterized by fatigue, weakness and tiredness, which are associated with reduced synthesis of adenosine triphosphate (ATP) as a result of mitochondrial dysfunction.

PBMT lowered the inflammatory response both in *in-vivo* and *in-vitro* studies; neuronal degeneration is elicited by the inflammatory and neurotoxic mediators.

PBMT was found to reduce oxidative stress in AD animal models.

PBM should be used as part of a comprehensive treatment plan, and individuals should seek guidance from healthcare professionals before considering PBM for neurological conditions.

PBM has been associated with improvements in cognitive function, reduction in post-concussion symptoms and accelerated recovery in individuals with mild to moderate TBI.

PBMT could be helpful in patients who are non-responsive to conventional pharmacological therapy.

Alzheimer's disease (AD) is considered a progressive neurodegenerative disease [1,2] and a severe age-related (especially in the elderly population) neurological disease [3], which is characterized by the reduction in cognitive functions like learning, memory, decision making, language abilities and motor activities [4,5]. AD is currently an incurable disease that may cause an immense economic burden on patients and society [6]. The major pathological marker involved in this neurodegenerative disease is the abnormal deposition of beta-amyloid (Aβ), which is an extracellular amyloid plaque originating from the amyloid protein precursor [^7-9^]. Mitochondrial dysfunction results from the reduced activity of cytochrome c oxidase (CCO) and from the imbalance between the mitochondrial fusion and fission protein [10]. Neuronal inflammation is characterized by microglial and astrocyte cell activity [4], induction of heat shock protein (HSP) [6] and cyclo-oxygenase pathway (COX) and NFκB stimulated neuritis [11]. Oxidative stress resulting from increased levels of reactive oxygen species (ROS) [^12-14^], and reduction in the synthesis of adenosine triphosphate (ATP) [15,16], and the expression of intracellular neurofibrillary tangles as mentioned in Figure 1 respectively [17].

**Figure 1.**
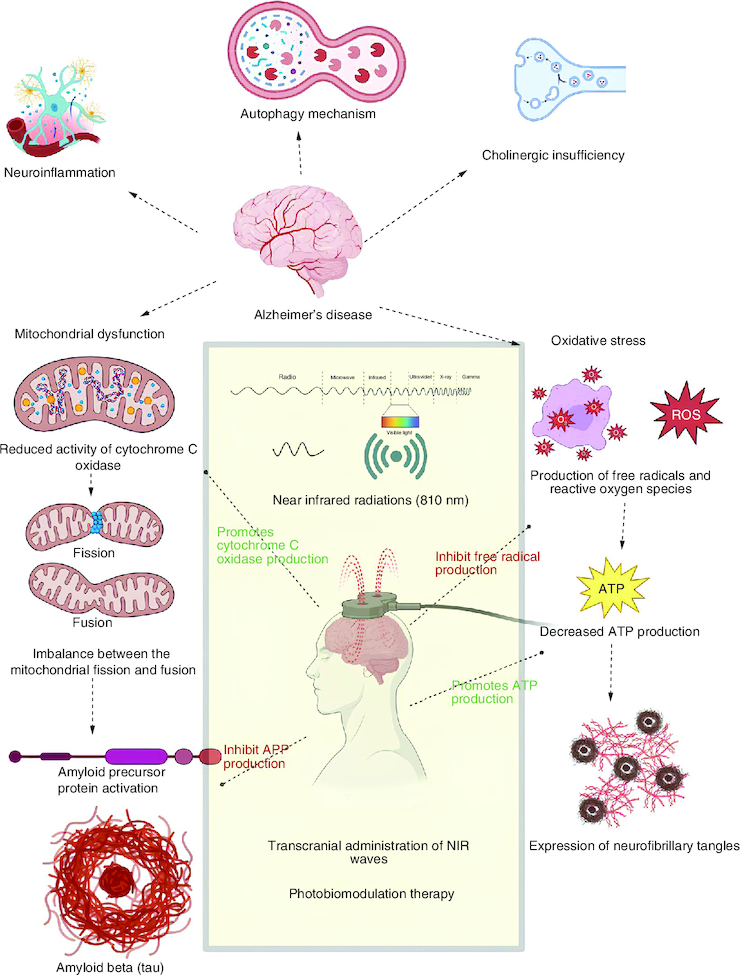
Photobiomodulation approach in the pharmacotherapy of Alzheimer's disease. The major pathophysiological mechanisms of AD involving the deposition of beta amyloid plaques and formation of neurofibrillary tangles, along with the role of photobiomodulatory therapy, are explained in the following figure.

Photobiomodulation (PBM), also known as low-level laser therapy (LLLT) or phototherapy, is a medical and therapeutic technique that uses specific wavelengths of light to stimulate, heal or regenerate tissue [4,6], which becomes one of the innovative and promising non-pharmacological therapy for a wide range of neurological and psychological disease [8]. Photobiomodulation therapy (PBMT) describes the therapeutic potential of red and near-infrared light in enhancing the healing mechanism, relieving pain and inflammation and preventing neuronal tissue from damaging and necrosis [18,19]. Transcranial PBM (tPBM) is a non-invasive therapy that works by passing low-level laser light or near-infrared light through the transcranial part of the head into the brain [6,20]. PBM causes a reduction in amyloid plaque formation by destroying the necessary precursor protein (APP) [21,22]. PBM restores mitochondrial homeostasis through the activation of cytochrome c oxidase and downregulation of nitric oxide (NO) [23]. PBMT has shown its anti-inflammatory potential by modulating the COX pathway, downregulation of heat shock protein and transcription of pro-inflammatory cytokines [24,25], modulating the release of ROS as antioxidant property [^26-28^].

Near infrared light (NIR) refers to light with wavelengths in the near-infrared spectrum, which falls within the range of 700–1400 nanometres. Red/NIR light possesses an intriguing ability to alter cell membrane polarization, triggering the influx of calcium ions and prompting the release of substances from the endoplasmic reticulum [29]. NIR light has been studied for its potential therapeutic effects on various medical conditions.β-amyloid is a protein that can aggregate and form plaques in the brains of individuals with Alzheimer's disease. These plaques are associated with the neurodegenerative process seen in the disease. Microglia are a type of immune cell found in the central nervous system. In the context of Alzheimer's disease, microglia can become activated and contribute to inflammation and neuronal damage. ‘Microglial toxicity’ likely refers to the harmful effects of activated microglia on neurons. NIR light may have the potential to protect neurons from damage and enhance their survival in the presence of Aβ and activated microglia. PBMT is used for the biosynthesis of ATP by the upregulation of cAMP and light-sensitive oxidative phosphorylation [30,31]. There also lies important evidence that tPBM shows an improvement in cognitive function through the modulation in brain neurological function [32,33]. This evidence is obtained from the electroencephalography (EEG) performed on the AD patient after the series of treatments with near-infrared light (810 nm) [^34-36^]. However, there is not currently a defined approach to using it for AD. The expert panel reviewed and discussed in-depth the mechanisms of action in amyloid plaque reduction, mitochondrial homeostasis, ATP production, anti-inflammatory effect and oxidative stress in order to direct its appropriate use. The conclusion was made based on a survey of the literature and clinical knowledge.

## Mechanism of PBM in AD management

### Role of PBM in amyloid plaque reduction

PBMT was found to reduce the deposition and overload of β-amyloid in the brain, especially in AD [1,^37-39^]. PBMT potential role in the management of AD has been inscribed in Table 1 respectively. Amyloid plaque is an extracellular toxin that originates from the Aβ protein [7], which is found to be the primary cause of cognitive dysfunction. Hence, the removal of amyloid plaque was found to be the main target in Aβ-mediated AD; however, the blood–brain barrier was the major barrier in delivering pharmaceutical medicament into the brain for the management of AD [40]. PBM involves the passing of low-level laser (600–700 nm) or near-infrared light (760–1200 nm) onto the transcranial position enhances the removal of deposited Aβ [20,22,41], and also results in the reduction of beta-amyloid production by inducing changes in the genetic expression of BACE1 (beta site APP-cleaving enzyme) and cathepsin B enzymes, which splits APP and is responsible for the production of Aβ [42,43], Another mechanism involved here is PBM mediated inactivation of JNK3 that leads to decreased endocytosis and dephosphorylation of synaptic AMPA (alpha amino-3-hydroxyl-5-methyl-4-isoxazolepropionic acid) receptors, which results in the further reduction of amyloid deposition [3]. Similarly, the studies showed that 1070 nm light depletes the Aβ deposit [3,4]. Thus, it has a proven effect in the improvement of cognitive functions like memory [44] learning in mice models with AD [21,45].

**Table 1.** Photobiomodulation approach in the pharmacotherapy of Alzheimer's disease.

S. No.	Aspect	Potential effects of PBM in AD	Ref.
1.	Inflammation reduction	PBM may reduce brain inflammation, a contributing factor in AD	[102]
2.	Cellular energy enhancement	PBM might enhance cellular metabolism and brain cell function	[103]
3.	Neuronal protection	PBM may have neuroprotective effects, supporting neuron survival	[104]
4.	Increased blood flow and oxygenation	PBM has been shown to improve blood flow and oxygenation in the brain	[105]
5.	Reduction of oxidative stress	PBM may help reduce oxidative stress associated with neurodegeneration	[104]

PBM can promote vasodilation and improve blood flow to the brain. Adequate blood flow is essential for the clearance of metabolic waste products, including amyloid beta, from the brain. Improved blood flow may facilitate the removal of amyloid plaques. PBM has anti-inflammatory properties, and chronic neuroinflammation is a hallmark of AD. By reducing inflammation in the brain, PBM may slow down the progression of the disease and potentially aid in the clearance of amyloid plaques. PBM may have a neuroprotective effect by promoting the survival and health of neurons. This can potentially reduce neuronal damage caused by amyloid plaques and other pathological processes associated with AD. Microglia are immune cells in the brain that play a role in clearing amyloid beta plaques. PBM may help modulate microglial activity, potentially enhancing their ability to phagocytose (remove) amyloid plaques. It is important to emphasize that while there is some promising preclinical and clinical research on the potential benefits of PBM in AD, the results are not yet conclusive. The field is still evolving, and more studies that are rigorous are needed to establish the safety and efficacy of PBM as a treatment for amyloid plaque reduction and AD [46].

### Role of PBM in maintaining mitochondrial homeostasis

Mitochondrial dysfunction is one of the pathological markers involved in neuronal dysfunction that leads to the development of AD [1,47], which is characterized by impaired mitochondrial dynamics and mitochondrial fragmentation [6]. Mitochondrial dysfunction increases the probability of stress response and transcription factor ATF4, which causes a modification of the CCO enzyme and induces morphological changes in the mitochondria, which results in reduced mitochondrial activity and glucose metabolism [18,48]. Mitochondrial dysfunction is elicited by the imbalance between the mitochondrial fusion proteins, like Opa1, Mfn1 and Mfn2 (which are decreased) and fission proteins like Drp1, Fis1, Mff and Mief (which are elevated) [^49-51^] and also includes mitochondrial enzymes provoked metabolic dysfunctions [52] are also involved in the development of AD [53,54]. PBMT was known to reinstate the mitochondrial activity and to maintain the mitochondrial homeostasis [42,55]. PBM with transcranial low-level laser therapy helps in balancing the ratio between mitochondrial fusion and fission protein through a shift in the mitochondrial dynamics and modification of antioxidant levels [6,56,57]. PBMT exhibits its mechanism as a stimulator of cytochrome c oxidase enzyme [1,18], which is a unit (IV) of the mitochondrial respiratory chain responsible for glucose metabolism and ATP synthesis [41,58]. NO was found to block the activity of cytochrome c oxidase enzyme [59], and hence, the PBM plays a significant role in disaffiliating the inhibitor NO, thereby increasing the availability of CCO enzyme in maintaining the mitochondrial membrane potential, oxygen consumption, glucose metabolism and ATP synthesis [19,60]. In addition, PBM was found to stimulate CCO [61] enzyme by activation of the cAMP/PKA signaling pathway, which in turn leads to the activation of SIRT1 that enhances the functional ability of mitochondria in AD [42]. Henceforth, it was observed that PBMT brings back mitochondrial homeostasis from mitochondrial dysfunction in AD models. The study was conducted by Amaroli *et al.* show the effects of PBM using a 980 nm diode laser on mitochondrial activity and the production of ROS. It likely presents findings suggesting that PBM with this specific laser can enhance mitochondrial function and reduce ROS levels. This implies potential therapeutic applications for conditions involving oxidative stress and mitochondrial dysfunction [62]. The study conducted by Golovynska *et al.* investigates the differential effects of near Infrared, visible, and ultraviolet (UV) light on the upregulation of ROS through the activation of photoreceptors within mitochondrial complexes in normal, immune, and cancer cells. The research aims to compare how these different types of light impact ROS production in various cell types [63].

### PBM-mediated ATP biosynthesis

AD is the most acquired neurodegenerative disease and the major cause of depression and dementia in an elderly population [7], which is characterized by fatigue, weakness, and tiredness, which are associated with reduced synthesis of ATP because of mitochondrial dysfunction [6]. Transcranial PBM (tPBM) or transcranial low-level laser therapy was found to promote ATP biosynthesis [6,53], cerebral blood flow, oxygen availability and consumption [64], and in the functional recovery of damaged human neuronal progenitor cells by maintaining the mitochondrial homeostasis [15,16], and through the PBM mediated increased CAMP level, oxidative phosphorylation and activation of PKA and SIRT1 signaling pathway [42]. Therefore, it paves the silver line in improving the symptoms associated with AD.

PBM has been shown to influence ATP biosynthesis within cells. ATP is often referred to as the ‘energy currency’ of cells because it provides the energy needed for various cellular processes. PBM can stimulate ATP production through several mechanisms: one of the key mechanisms through which PBM influences ATP production is by activating cytochrome c oxidase, a component of the electron transport chain in mitochondria. Cytochrome c oxidase plays a crucial role in the final step of cellular respiration, where oxygen and electrons are used to generate water and ATP. PBM enhances the activity of cytochrome c oxidase, which increases the efficiency of ATP production [65].

PBM can improve the flow of electrons along the electron transport chain in mitochondria. This improved electron transport allows for the generation of a proton gradient across the inner mitochondrial membrane, which is essential for ATP synthesis. PBM can increase the mitochondrial membrane potential, which is the electrical potential difference across the inner mitochondrial membrane. A higher membrane potential is associated with increased ATP production. PBM can specifically stimulate the activity of complex IV (cytochrome c oxidase), which is the enzyme responsible for transferring electrons to oxygen and driving the synthesis of ATP in the mitochondria. PBM has been shown to upregulate the expression of genes associated with mitochondrial biogenesis. This means that PBM can promote the generation of new mitochondria, increasing the overall capacity for ATP production. By enhancing these mitochondrial processes, PBM helps cells generate ATP more efficiently. This can have various benefits, including improved cellular function, tissue repair and energy production. In the context of various medical applications, such as wound healing, muscle recovery, or neurological conditions, the increased ATP production from PBM may contribute to the therapeutic effects observed [66].

### PBM-mediated anti-inflammatory effect

Inflammation is another criterion that is responsible for the development of the pathological progression in AD [7]. It has been observed that the PBMT lowered the inflammatory response both in *in vivo* and *in vitro* studies; neuronal degeneration is elicited by the inflammatory and neurotoxic mediators [1,67,68]. Additionally, the microglial cell activity and astrocyte activity contribute to the clearance of Aβ through the process of phagocytosis [4,69]. PBMT using 1070 nm light causes conformational morphological changes in the microglia in the cortex that lead to the activation of microglia cells, which enhances the capacity of the microglial phagocytosis process [41,70]. It was also found that astrocytes were involved in the phagocytosis and degradation of beta-amyloid in the cortex of AD mice model that is induced by the 1070 nm light therapy [4,71]. PBMT has the ability to shift the microglial phenotype from an M1 pro-inflammatory type to an M2 anti-inflammatory type. This change in microglial behaviour suggests that PBMT may have immunomodulatory effects, potentially reducing inflammation and promoting tissue healing in various conditions [18,^72-74^]. The HSP signaling pathway was also found to be involved in the inflammatory response, which is mediated through the accumulation of beta-amyloid in return for HSP activation [6,75]. In order to counteract the HSP mechanism [76], low laser light therapy elicits the anti-inflammatory role by decreasing the beta-amyloid plaque formation and decreasing the expression of inflammatory markers in the AD mice model [6,77]. The stability of microglia surrounding the amyloid plaque can be indicated using PBM with a NIR probe named CRANAD-3 at an imaging dose (2 mg/kg) [7]. Chronic infection with microorganisms like bacteria and viruses can lead to chronic inflammation as a part of the innate immune defense mechanism, which is mediated through the COX-1,2 pathway, NFκB depends on intracellular nucleus pro-inflammatory gene transcription process, and ATP-Sensitive K+ Channel/p38-MAPK signalling pathway [18,78]. However, PBMT plays an important role in inhibiting the COX enzymes, inhibiting the mediated gene transcription, and modulating the level of pro-inflammatory cytokines (TNF-α) [79] and anti-inflammatory cytokines [80], thus reducing the inflammatory response [81]. This is how the PBMT elicits the anti-inflammatory property in the overall improvement of neurological functional ability in AD individuals [82,83]. This study conducted by Stepanov *et al.* examines the therapeutic potential of NIR light for Alzheimer's disease. The research findings suggest that NIR light can reduce microglial toxicity induced by Aß; a protein associated with Alzheimer's disease, and promote the survival of neurons. The paper likely delves into the mechanisms underlying these effects, shedding light on how NIR light therapy may hold promise as a treatment approach for Alzheimer's disease [84].

### Role of PBM in modulating the oxidative stress

Oxidative stress is yet another criterion for the progression in the pathology of AD, which is characterized by the imbalance between the liberation of ROS and antagonizing their effect in the body, which are closely connected with neuronal degeneration [1,85]. Various reasons were illustrated for the development of oxidative stress, including mitochondrial impairment, elevated heavy metal levels, and neuronal inflammation [12,86,87]. Neurodegeneration associated with oxidative stress is mainly through the contribution of accumulation of beta-amyloid, hyperphosphorylation, and impairment of synapses and neurons [18,88]. PBMT was found to reduce oxidative stress [89,90] in AD animal models [6,91]. tPBM with near-infrared light (810 nm) elicits elevated antioxidant enzymes with lowered oxidative stress cytokine (iNOS- inducible nitric oxide synthase) that is responsible for the increased liberation of reactive oxygen species/reactive nitrogen species known as peroxynitrite, which in turn responsible for the oxidative stress [26,92,93]. In such conditions, the PBMT attenuates the so-formed peroxynitrite, thereby reducing the oxidative stress [18,94]. It was also known that the PBMT was found to elevate the ROS in the positive concept of provoking the cytoprotective, antioxidant, and anti-necrotic effects on the neurons [6,30,95]. Additionally, tPBM also enhances cerebral blood flow through NO mediated vasodilation [19,58]. PBMT is also known to be beneficial in chronic, unpredictable, mild stress-induced depression by altering the glutamatergic dysfunction by regulating GLT-1 mRNA and protein levels via the Akt/NF-κB signaling pathway [96].

In the context of neurological conditions, PBM has garnered significant interest due to its potential to modulate neural activity, promote neuroprotection, reduce inflammation, and enhance tissue repair. Basic research studies using animal models of neurological conditions have demonstrated that PBM can exert neuroprotective effects. It can enhance the survival of neurons and protect them from damage caused by various insults, such as oxidative stress, excitotoxicity and inflammation. PBM's neuroprotective properties make it a promising therapeutic approach for conditions like traumatic brain injury (TBI) and stroke. Inflammation plays a crucial role in the progression of many neurological disorders. PBM has been shown to modulate the inflammatory response in the brain. It can reduce the production of pro-inflammatory cytokines and promote the release of anti-inflammatory factors, contributing to a more favourable environment for neurological recovery. PBM has been found to stimulate the formation of new neurons (neurogenesis) and promote the growth of synapses (synaptogenesis) in the brain. These effects are particularly relevant in conditions where neuronal loss occurs, such as neurodegenerative diseases. By promoting neurogenesis and synaptogenesis, PBM may support neural repair and functional recovery. PBM can influence neuronal activity by altering ion channel function and neurotransmitter release. It may have both inhibitory and excitatory effects on neural circuits, depending on the specific parameters used. This ability to modulate neural activity could have implications in the treatment of conditions characterized by abnormal brain rhythms, such as epilepsy.

The paper titled ‘Bright light therapy: improved sensitivity to its effects on rest-activity rhythms in Alzheimer patients by application of nonparametric methods’, authored by Van Someren and colleagues in 1999 explores the use of bright light therapy in Alzheimer's patients and how nonparametric methods can enhance the sensitivity of detecting its effects on their rest–activity rhythms. The key findings of this study likely suggest that bright light therapy may have a positive effect on the rest-activity rhythms of Alzheimer's patients, and the use of nonparametric methods improved the sensitivity of detecting these effects. This could have implications for the treatment and management of sleep disturbances and circadian rhythm disruptions commonly seen in Alzheimer's disease [97].

The paper titled ‘Effect of light therapy upon disturbed behaviours in Alzheimer patients’, authored by Rheaume and colleagues in 1998 investigates the impact of light therapy on behavioural disturbances in individuals with Alzheimer's disease. The study likely involved a group of Alzheimer's patients who were exposed to light therapy as an intervention. Light therapy typically involves the controlled exposure of individuals to specific types and intensities of light, often mimicking natural daylight patterns. The researchers would have assessed changes in the participants' behavior following the light therapy sessions. The findings of this study would provide insights into whether light therapy could serve as a non-pharmacological approach to managing behavioural disturbances in Alzheimer's patients. Such interventions are valuable because they can help improve the quality of life for both patients and their caregivers without relying on medications, which may have side effects or interactions with other treatments [98].

The paper titled ‘The effect of 40-Hz light therapy on amyloid load in patients with prodromal and clinical Alzheimer's disease’, authored by Ismail and colleagues in 2018 examines the impact of 40-Hz light therapy on the accumulation of amyloid in individuals with prodromal (early-stage) and clinical (established) Alzheimer's disease. The paper likely reports the outcomes of their investigation, including any changes observed in amyloid load in participants with prodromal and clinical Alzheimer's disease after receiving 40-Hz light therapy. It could suggest that this therapy has the potential to influence the pathological processes associated with Alzheimer's disease, particularly the accumulation of amyloid plaques. Understanding the effects of 40-Hz light therapy on amyloid load is significant because it could open up new avenues for non-pharmacological interventions in Alzheimer's disease treatment and potentially contribute to the development of therapies aimed at slowing down the progression of the disease [99].

## Other clinical applications

Traumatic brain injury (TBI) is a significant public health concern, and there is a need for effective treatments to improve outcomes. Clinical studies exploring PBM for TBI have shown promising results and mentioned in Table 2, respectively. PBM has been associated with improvements in cognitive function, reduction in post-concussion symptoms, and accelerated recovery in individuals with mild to moderate TBI [100]. It is believed that PBM's neuroprotective and anti-inflammatory effects contribute to these beneficial outcomes. Stroke is another neurological condition where PBM has been investigated. Animal studies have shown that PBM can enhance neurogenesis, reduce brain damage and improve functional recovery after a stroke. Clinical trials in stroke patients have demonstrated potential benefits, including improved motor function and enhanced rehabilitation outcomes. Although research in this area is still in its early stages, preclinical studies have suggested that PBM might have neuroprotective effects in neurodegenerative diseases such as Alzheimer's and Parkinson's disease. It may help reduce neuronal damage, slow disease progression, and improve cognitive function. PBM has been studied for its analgesic effects in various pain conditions, including neuropathic pain. By reducing inflammation and promoting tissue repair, PBM may help alleviate pain and improve the quality of life in patients with neuropathic pain. Some research has explored the potential antidepressant effects of PBM. While the mechanisms are not fully understood, PBM's ability to influence brain activity and promote neuroplasticity might contribute to its mood-modulating effects. It is important to note that despite the promising findings, the field of PBM in neurological conditions is still evolving and many questions remain to be addressed. Factors such as optimal treatment parameters (e.g., wavelength, dosage and duration), long-term safety and individual variability in treatment response need further investigation. Large-scale, well-designed clinical trials are necessary to establish PBM as a standard therapeutic approach for specific neurological disorders. Additionally, PBM should be used as part of a comprehensive treatment plan, and individuals should seek guidance from healthcare professionals before considering PBM for neurological conditions [101].

**Table 2.** Various clinical applications of photobiomodulation.

S. no.	Clinical application	Description	Ref.
1.	Neurological disorders	Some studies suggest potential benefits in treating neurodegenerative disorders like Alzheimer's and Parkinson's disease by promoting neuroplasticity and reducing oxidative stress	[106]
2.	Wound healing	PBM can accelerate the healing process of various wounds, including surgical wounds, diabetic ulcers and burns. It promotes cell proliferation, angiogenesis and collagen synthesis	[107]
3.	Pain management	It is used to alleviate acute and chronic pain conditions, such as musculoskeletal pain, arthritis, neuropathy, and fibromyalgia. PBM helps reduce inflammation and enhances the release of endorphins	[108]
4.	Oral health	PBM is employed in dentistry to accelerate post-operative healing, manage oral ulcers and treat temporomandibular joint disorders	[109]
5.	Dermatology	In dermatology, PBM is used to treat conditions like psoriasis, acne and scars by stimulating cellular repair and modulating inflammation	[110]
6.	Ophthalmology	It has shown promise in treating certain eye conditions, like age-related macular degeneration and retinitis pigmentosa	[111]
7.	Cognitive enhancement	Research is ongoing regarding the potential of PBM for improving cognitive function and mental health conditions	[112]
8.	Veterinary medicine	It is used in animals to promote wound healing, manage pain and treat various conditions, similar to its human applications	[113]

## Conclusion

From the above-mentioned information, it is evident that the PBMT is projected as a tool of innovative and non-invasive therapy using the light for the management of AD and thereby evoking cognitive improvements such as learning, memory, decision making and language ability through the destruction of beta-amyloid plaque, restoration of mitochondrial function, anti-inflammatory and antioxidant mechanism in patient with AD. The PBMT could be helpful in patients who are non-responsive to conventional pharmacological therapy. However, this therapy is found to provide significant aid in the management of AD when introduced into the medical field. However, it requires various intensive research to be conducted for further conclusion.

## Future perspective

The effectiveness and safety of PBM (photobiomodulation) in Alzheimer's disease necessitate further rigorous scientific exploration. To definitively establish its therapeutic potential, future research should encompass broader patient cohorts, longer-term evaluations and rigorous randomized controlled trials.

It is of paramount importance to ascertain the ideal parameters for PBM in the treatment of Alzheimer's disease. This encompasses the identification of the most efficacious wavelengths, energy densities and treatment durations. Standardizing these parameters can enhance the consistency and reproducibility of the treatment.

Customizing PBM protocols according to the unique characteristics of individual patients, such as their disease stage, genetic profile, and therapy response, holds the potential to enhance its efficacy. Personalized medicine strategies may see increased adoption in the future, allowing for more tailored and effective treatments. Integrating PBM with other therapeutic modalities, such as pharmaceutical interventions or cognitive rehabilitation, has the potential to produce synergistic effects in the management of Alzheimer's disease. Exploring these combined treatment approaches through research may pave the way for more comprehensive and effective strategies for managing the condition.
